# C‐Jun N‐terminal kinase signalling pathway in response to cisplatin

**DOI:** 10.1111/jcmm.12908

**Published:** 2016-07-04

**Authors:** Dong Yan, GuangYu An, Macus Tien Kuo

**Affiliations:** ^1^ Department of OncologyBeijing Chao‐Yang Hospital Affiliated with Capital Medical UniversityBeijingChina; ^2^ Department of Translational Molecular PathologyThe University of Texas MD Anderson Cancer CenterHoustonTXUSA

**Keywords:** C‐Jun N‐terminal kinase signalling pathway, cisplatin, apoptosis, resistance

## Abstract

Cisplatin (cis diamminedichloroplatinum II, cDDP) is one of the most effective cancer chemotherapeutic agents and is used in the treatment of many types of human malignancies. However, inherent tumour resistance is a major barrier to effective cisplatin therapy. So far, the mechanism of cDDP resistance has not been well defined. In general, cisplatin is considered to be a cytotoxic drug, for damaging DNA and inhibiting DNA synthesis, resulting in apoptosis *via* the mitochondrial death pathway or plasma membrane disruption. cDDP−induced DNA damage triggers signalling pathways that will eventually decide between cell life and death. As a member of the mitogen‐activated protein kinases family, c‐Jun N‐terminal kinase (JNK) is a signalling pathway in response to extracellular stimuli, especially drug treatment, to modify the activity of numerous proteins locating in the mitochondria or the nucleus. Recent studies suggest that JNK signalling pathway plays a major role in deciding the fate of the cell and inducing resistance to cDDP‐induced apoptosis in human tumours. c‐Jun N‐terminal kinase regulates several important cellular functions including cell proliferation, differentiation, survival and apoptosis while activating and inhibiting substrates for phosphorylation transcription factors (c‐Jun, ATF2: Activating transcription factor 2, p53 and so on), which subsequently induce pro‐apoptosis and pro‐survival factors expression. Therefore, it is suggested that JNK signal pathway is a double‐edged sword in cDDP treatment, simultaneously being a significant pro‐apoptosis factor but also being associated with increased resistance to cisplatin‐based chemotherapy. This review focuses on current knowledge concerning the role of JNK in cell response to cDDP, as well as their role in cisplatin resistance.

## Introduction

Cisplatin was first created in the mid‐19th Century and discovered by Michel Peyrone. But until the 1960s, scientists started getting interested in its biological effects. Cisplatin went into clinical trials for cancer therapy in 1971. Up to now, it had already widely used and still be used despite many newer chemotherapy drugs developed over the past decades.

Cisplatin (cisdiamminedichloro‐platinum II, cDDP)‐based chemotherapy is the mainstay regimens for many types of human malignancies such as testicular cancer 1, lung cancer 2, breast cancer 3, ovarian cancer 4, bladder cancer 5 and so on. However, intrinsic or acquired drug resistance during the course of the treatment is the major limitation of its usage 6, 7, 8. The mechanisms involved in cDDP resistance include reduced drug uptake, increased drug inactivated and DNA adduct repair. Therefore, the mechanisms of resistance to cDDP are generally multifactorial and complicated.

Our previous studies has been elucidated that transport impairment is an important mechanism of resistance to cDDP. The major import transporter for platinum‐based antitumour agents including cisplatin and carboplatin is the high‐affinity copper transporter 1 (hCtr1), and efflux transporters are the two p‐type ATPase, ATP7A and ATP7B 9, 10. While mutations in these transporters are infrequent, reduced hCtr1 expression and elevated ATP7A and ATP7B expression are frequently associated with cDDP resistance. Furthermore, recent studies demonstrate that DNA damage‐mediated signals are considered to play core role in limitation of cisplatin‐based chemotherapy. DNA is the principal cellular lethal target of cDDP by forming DNA‐Pt cross‐link adducts 11, 12, 13, constituting 85–90% of total cDDP lethal effects 14, 15, 16. Formation of Pt‐DNA cross‐links distorts the structure of DNA and interferes with normal transcription and DNA replication mechanisms 17, 18, 19, 20. Limited the extent of DNA damage or increased repair of tolerance to these DNA lesions *via* activating and transducing DNA damage signals are important mechanisms of cDDP resistance.

DNA damage triggers signals to induce resistance include loss of damage recognition, loss of p53 function, overexpression of anti‐apoptotic bcl‐2 and interference in caspase cascade activation. All these events involve in activation of the c‐Jun N‐terminal kinase (JNK) pathway. Hyperactivation of the JNK proteins has been reported in multiple cancer cell lines and tissue samples, which evokes downstream pathway of drug resistance to escape apoptosis in a range of human cancers, including hepatocellular carcinoma, lung adenocarcinoma, colon carcinoma, or trip‐negative breast cancer 21, 22, 23, 24, 25. In this communication, we will present evidence illuminating the importance of JNK pathway in cDDP‐induced cytotoxicity and mechanisms thereof.

## c‐Jun N‐terminal kinase

Stress‐activated protein kinases (SAPK)/JNKs are members of the mitogen‐activated protein kinase (MAPK) family and are activated by cellular environmental stresses, inflammatory cytokines and growth factors 26, 27. Upon stimulations, SAPK/JNK then translocate to the nucleus where it regulates the activity of multiple transcription factors that are involved in oncogenic transformation, growth, differentiation, cell survival and death. In mammalian cells, JNK derives from three genes: *jnk1*,* jnk2* and *jnk3*. JNK1 and JNK2 are ubiquitously expressed in cells and tissues, both of which contribute to the majority of JNK activity. It had been demonstrated that complete genetic disruption of both *jnk1* and *jnk2* alleles in mouse embryos is lethal 28, 29. However, different JNK isoforms have distinct functions and the underlying molecular mechanisms for the discrepancy between the JNK1 and JNK2 isoforms is unknown. Indeed, JNK1 and JNK2 also have opposing biological functions in some cases 30, 31. It is generally considered that the function of JNK depends on the cell type, nature of the stimulus, duration of its activation and activity of other signalling pathways. Recent studies suggest that JNK2 interferes with JNK1 activation in response to the extracellular stimuli, including tumour growth factor (TNF)‐α, UV and some drugs, to regulate cellular survival or apoptosis 32, 33. In this review, we will attempt to clarify the molecular mechanisms underlying the different biological functions of JNK isoforms response to cDDP.

## JNK pathways and networks

Stress‐activated protein kinases/JNK are members of the MAPK family involved in sequential activation of a kinase cascade responsible for environmental stress and cytokines (*e.g*. TNF and growth factors) in many physiological and pathological processes. Triggered DNA damage signalling pathways have been demonstrated to be important for the mechanism of resistance to cisplatin. Meanwhile these signals are delivered by G‐proteins, tumour necrosis factor receptor associated of adaptor proteins and small GTPases of the Rho family (Rac, Rho, cdc42) to activate the JNK pathway cascade. Activation process starts with the activation of MAPK kinase kinases (MAPKKKs), then, phosphorylated MAPKKKs activate the downstream MAPKK which in turn phosphorylate and activate MAPK through cascades of signalling pathway. MAP2 Kinases MKK‐4, MKK‐3, MKK‐6 and MKK‐7 are involved in the activation of the JNK pathway. Downstream molecules are activated by JNK including c‐Jun, ATF2, ELK1, SMAD4, p53 and HSF1, and inhibited by JNK including NFAT4, NFATC1 and signal transducer and activator of transcription 3 (STAT3) 34. Furthermore, multiple transcription factors including JunD, RXRα, RARα, activator protein‐1 (AP‐1) and c‐Myc are regulated by JNK phosphorylation. Additional substrates include mitochondrial proteins (SH3 homology associated BTK binding protein (Sab)), metabolic regulators (insulin receptor substrate 1), microtubule‐associated proteins (stathmin) and cell death pathway proteins (*Bcl‐2*, Bcl‐XL, Bid, Bim, Bad and Bax). c‐Jun N‐terminal kinase‐interacting proteins (JIP) acts as a molecular scaffold protein to form a multiprotein complex with other JNK apoptotic cascade elements 35, 36. Whether JNK has a pro‐survival or a pro‐death role is may be explained by the selective interaction of JNK protein kinase isoforms with different transcription factors.

## JNK pathways is involved in cisplatin‐induced cell death

Binding of cDDP to genomic DNA is largely responsible for induction of cell death, but only 5–10% of covalently bound cell‐associated cDDP is found in the DNA fraction, whereas 75–85% of this drug binds to proteins 37. Recently, it has been known that the cytostatic/cytotoxic effects of cDDP occur *via* both nuclear and cytoplasmic signalling pathways involving induction of apoptosis. All these events activate a network of signalling pathways that can activate JNK signalling cascades, determining the final outcome in cells treated with cDDP.

### DNA damage

cDDP−induced DNA adducts comprise the bulk of cisplatin‐induced nuclear lesions. DNA damage recognition proteins recognize of DNA distortions and transduce DNA damage signals to the downstream effectors resulting in apoptosis response for cDDP 38. Meanwhile, an enhanced rate of adducts repair will attenuate the apoptotic process 39. Thus, both pro‐survival and pro‐apoptotic signals are activated simultaneously following cisplatin exposure, balance of the process determines the final fate of the cell in response to cisplatin.

### P53 and JNK pathway

With its functions in cell‐cycle arrest and apoptosis, p53 is an essential component in the response to DNA damage 40. DNA damage induces the expression of PIDD (p53‐induced death domain protein), which contains a p53 consensus DNA binding sequence and binds to RAIDD (RIP‐associated ICH1/CED3‐homologous protein with death domain) leading to the activation of caspase‐2. Previous study revealed that p53 protein levels are increased drastically, and eventually lead to a quick accumulation of p53 in cDDP‐treated cells. In turn, p53 is activated by phosphorylation of its N‐terminal domain as a transcription regulator which results in the transcription of p53 target genes in these cells. These results suggest that the critical event leading to cell‐cycle arrest or apoptosis in response to DNA damage in the cellular toxic effects of cisplatin is the p53 phosphorylation. A known upstream event inducing p53 activation is the JNK pathway activation in which regulates the stability and transcription of the p53. c‐Jun N‐terminal kinase activity requires MAPK kinase which phosphorylates MKK4/7. MKK4/7, in turn, phosphorylates JNK on residues 183 and 185. Activated JNK phosphorylates its substrates, c‐Jun, ATF2 and p53. Nevertheless, JNK1 and JNK2 had opposite effects in regulating p53, which appears to participate in a feedback mechanism with JNK to facilitate this apoptotic process. Several studies have shown that sustained activation of JNK1 down−regulates p53 expression during apoptosis, otherwise JNK2 binds p53 to protect its degradation and increases stability by competition with mdm2, leading to p53‐mediated apoptosis. Thus, JNK1 is a negative regulator and JNK2 is a positive regulator of p53 expression 41, 42.

### BCL‐2 family and JNK pathway

Another mechanism of cDDP‐induced cell death is due to the transduction of pro‐apoptotic *Bcl‐2* family. As a pro‐apoptotic member of the *Bcl‐2* family, Bim is a BH3‐only protein which function responds to DNA damage and provokes apoptosis. It had been reported that Bim is phosphorylated in a JNK‐dependent manner 43. c‐Jun N‐terminal kinase 1 and 2 also phosphorylate Bid, another BH3‐only pro‐apoptotic member, at Thr59 within the caspase cleavage site in PC‐3 prostate cancer cells. Then, phosphorylated Thr59 protects Bid from cleavage by caspase‐8, resulting in strong accumulation of the full‐length protein and apoptosis.

### ROS and JNK pathway

Apart from induction of DNA damage, the recent data has suggested that cDDP induces the formation of reactive oxygen species (ROS) that play a crucial role in cisplatin‐induced dose‐limiting toxicities. Indeed, *Bcl‐2* family proteins regulate ROS production under apoptotic conditions by inducing the mitochondrial permeability transition 44. Nevertheless, accumulation of ROS productions promote the activation of JNK and facilitate p53 function upon its release from Mdm2 24, 41, 42. In turn, activated p53 induces pro‐oxidant genes, which increases the level of ROS, further activating JNK, thus, amplifying p53 activity in contributing to apoptosis 45, 46, 47. Therefore, generation of ROS leads to DNA damage response and confers synthetic lethality upon p53 reactivation.

## JNK pathway plays an important role in cisplatin drug resistance

As one of the most central downstream kinases of Ras, JNK pathway plays a significant role in cell response to cDDP, and is associated with the induction of cell‐cycle arrest and apoptosis 48. In one research, inhibition of JNK activity by transfection with a dominant‐negative allele of JNK blocked cDDP‐induced apoptosis significantly in A2780 ovarian cancer cells 49. However, some reports are conflict. c‐Jun N‐terminal kinase signalling also contributes to cell survival signalling through inhibiting the phosphorylation of pro‐apoptotic proteins. Therefore, Inhibition of SAPK/JNK may sensitize human cancer cells to cDDP.

In a recent study, it was demonstrated that JNK activation contributes to the survival of 12 colon cancer cell lines. Transfection of dominant‐negative JNK1 blocked SAPK/JNK activation in colon cancer cell line HT29 and rendered it sensitive to oxaliplatin under hypoxia. Correlation between decreased activation of SAPK/JNK and cDDP‐induced apoptosis was confirmed on different tumour cell lines. Recently, the JNK inhibitor has been proposed as a promising antitumour agent that induces apoptosis of some tumour cells. Treating cells with JNK inhibitor CC‐401 resulted in increased sensitivity to chemotherapy 50.

**Figure 1:**
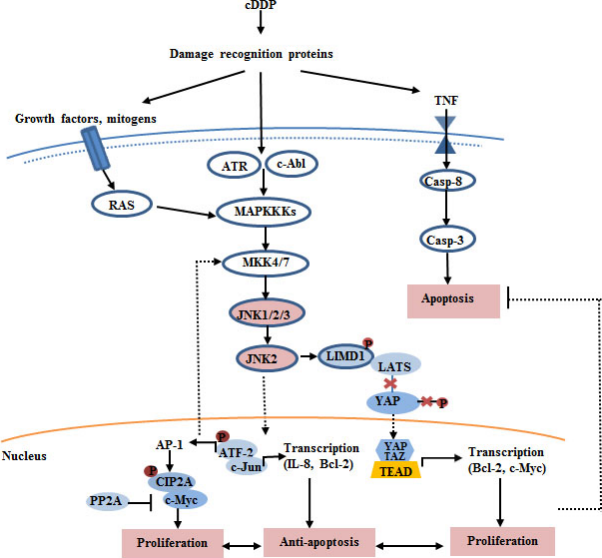
JNK pathway and cDDP resistance.

Although the molecular mechanisms that underlie the enhance sensitivity to cDDP‐induced apoptosis are poorly understood, recent studies suggested that JNK pathway is a double‐edged sword in cDDP treatment, simultaneously not only being a significant factor of cell death but also being associated with increased resistance to cDDP‐based chemotherapy *via* induction of cell survival 51. The molecular mechanism model is shown in Figure 1.

### Apoptotic pathways and cisplatin resistance

Apoptotic pathways alteration is one of the most important molecular mechanisms of cellular resistance to cisplatin. *Bcl‐2* is considered as an important anti‐apoptotic protein which controls cDDP‐induced programmed cell death process 52. Studies have found that *Bcl‐2* proteins are distributed not only in the mitochondrial outer membrane but also in the cell membrane, ER and nuclear membrane 53. ER‐localized *Bcl‐2* family anti‐apoptotic proteins such as *Bcl‐2* and *Bcl‐xL* suppress apoptosis in response to cDDP, including ER‐localized pro‐apoptotic proteins Bax/Bak and various BH3‐only proteins. Previous studies have found that Bax and Bak can combine with IRE1α on the cytoplasmic, then recruit ASK1, leading to activation of the pro‐apoptotic signalling kinase JNK and downstream pro‐apoptotic transcription factor c‐Jun. Although, JNK is known to regulate starvation‐induced autophagy through *Bcl‐2* phosphorylation, which results in the migration of *Bcl‐2* from the mitochondrial membrane and plays protective role in cDDP‐induced ROS formation, thereby inducing cDDP resistance and enabling cell survival 54, 55, 56, 57. In addition, JNK2 causes BiP up‐regulation and protection from death 58. Some study indicated that JNK2 has a specific role as a regulator of the cellular acidic compartment, preventing its accumulation in ER‐stressed cells undergoing autophagic flux involved in cancer cell responses to chemotherapy. However, Pietkiewic *et al*. 31 reported that JNK1 greatly accelerates apoptosis while it is impaired in presence of JNK2 implying oppositional roles for these isoforms in proteasomal inhibitors induced apoptosis. In a recent study, JNK inhibitor IX (JNKi), targeting JNK2, causes apoptotic DNA fragmentation along with G2/M arrest, phosphorylation of *Bcl‐2* family proteins, and activation of Bak caspase cascade. It is suggested that JNK2 is one of the potential therapeutic targets of cDDP resistance.

### DNA repair and cisplatin resistance

It has been demonstrated that the activation of a wide range of DNA damage recognition and repair enzymes in response to JNK signalling, mostly *via* the JNK activated transcription factors FOXO or AP‐1. cDDP induces AP‐1 activity by JNK‐dependent phosphorylation. Activated AP‐1 regulates the activities of 23 DNA repair and repair‐associated genes, such as MSH2, MLH1, XPA, RAD21, RAD50, GADD45 and ERCC1, providing a molecular basis for the enhanced DNA repair 59. In addition, JNK can also regulate the activity of ATF‐2 by phosphorylation in response to various stimuli such as cytotoxicity agents. Subsequently, activated ATF‐2 affects a series of target genes involved in cell survival processes and DNA repair activity by activation of MRN and ATM pathway 60. Another study showed that forkhead transcription factors of the O class 1 (FOXO1) formed a complex with JNK kinase during DNA damage in p53‐deficient cell line H1299 cells. Furthermore, when using JNK inhibitor suppressed the nuclear translocation of FOXO1 preventing its target gene expression. It is suggested that FOXO1 serving as a substrate, may be involved in JNK pathway‐mediated DNA repair response 61. Beside the mentioned above, activated Epidermal growth factor receptor (EGFR) signalling results in activation of IRF3 and transcription of IRF3‐dependent downstream gene *via* JNK pathway in DNA repair processes 62. NIH3T3 cells expressing EGFRvIII, a constitutively positive mutation, exhibited a high basal level of JNK activity. Meanwhile, these cells have a low rate of apoptosis with high level of JNK activity 63.

### Hippo pathway and JNK pathway

Acting downstream of Ras signalling, JNK signalling promotes Ras‐induced tumour growth and progression in imaginal tissue, and induces a 2.7‐fold cDDP resistance in human cells. As the Hippo pathway effector, YAP is also a direct substrate for JNK phosphorylation in a number of cell lines. Recent datum has been shown that in certain conditions JNK signalling promotes tissue growth by activation of Yki/YAP/TAZ, and indeed it was found that blocking JNK2 activity in clones expressing RasV12 strongly suppressed Yki/YAP/TAZ activation, thereby suppressing tissue growth 64. Some studies reveal that Myc is a target gene of Hippo‐YAP pathway 65, 66. Furthermore, negative feedback regulation from Myc to Yki/YAP/TAZ is suggested to balance growth 67. Myc expression correlates with resistance to cDDP in cell line models and animal models 68. In c‐myc‐transfected NIH3T3 cells, c‐myc expression has been associated with acquired drug resistance for five antitumour agents, including cDDP and doxorubicin *in vitro*. Down‐regulation of endogenous c‐myc expression, may increase sensitivity to cDDP in the cDDP‐resistant human small‐cell lung cancer cell line. Protein phosphatase 2A facilitates the proteolytic degradation of oncoprotein Myc and prevents malignant cell growth 69. Cancerous inhibitor of protein phosphatase 2A (CIP2A) is a recently identified human oncoprotein that stabilizes c‐Myc protein by inhibiting its degradation. Previous research has shown that JNK2 is a key regulator of CIP2A expression and regulates CIP2A transcription *via* ATF2 70. The most important implication of these findings is the impact of JNK2 involved in cisplatin drug resistance. On the other hand, STAT3, as the JNKs substrate, activity by phosphorylating the Ser727 residue is mediated by a constitutively active JNK2. Activation of Stat3 signalling is shown to be correlated with the resistance of cancer cells to cisplatin‐induced apoptosis, and suppression of STAT3 by siRNAs increases the chemotherapeutic sensitivity of cisplatin‐resistant non‐small‐cell lung cancer cells 71. In another word, JNK pathway, especially JNK2, plays a very important role in the final outcome of cell treatment with cDDP.

## Conclusions and future directions

It is believed that JNK signalling pathway activation is a major factor deciding the fate of the cell in response to cDDP. Because multiple signalling pathways are integrated in a very complex network and the final response depends on the balance of activity of several of these networks, the cell type, as well as proliferation and differentiation status of tumour cells.

Nowadays, these are the key problems that JNK inhibitors are not used in clinical therapy due to the need for JNK in normal cell maintenance as well as for tumour cell apoptosis. However, new generation of JNK inhibitors are being developed and required extensive tests *in vivo* validation. Therefore, the high selectivity JNK inhibitors might be important clinical application value in chemotherapy, and while planning clinical studies by evaluating JNK functions in both health and disease.

## Conflict of interest

We declare that there is no conflict of interest.
